# Cyber-dating abuse in young adult couples: Relations with sexist attitudes and violence justification, smartphone usage and impulsivity

**DOI:** 10.1371/journal.pone.0253180

**Published:** 2021-06-21

**Authors:** Rocío Linares, María Aranda, Marta García-Domingo, Teresa Amezcua, Virginia Fuentes, María Moreno-Padilla

**Affiliations:** Department of Psychology, University of Jaen, Jaen, Spain; University of Padova, ITALY

## Abstract

Technologies have become important for interaction in couples. However, in some cases, controlling and aggressive behaviors can occur in the context of virtual interactions in couples; this is known as cyber-dating abuse (CDA). Identifying factors linked to CDA, as perpetrator and victim, are relevant for its prevention; therefore, more research is needed in this novel field of study. To contribute to the literature, our first goal was to analyze the associations among certain risk factors for CDA perpetration and victimization of, i.e., sexist attitudes and violence justification, problematic smartphone usage and impulsivity; sex and age were also considered. The second goal was to study whether there were differences in direct aggression and control, from the perpetrator and victim perspectives, with consideration of the above-mentioned risk factors. Third, differences in the diverse range of control behaviors and direct aggression between women and men were explored. To this end, 697 young adults (aged between 18 and 35 years; 548 women) completed self-report questionnaires that allowed assessment of the above-mentioned variables. The results showed that, among the wide range of CDA behaviors, indirect ones such as control behaviors were the most common. The highest level of control was strongly associated with the inability to manage behaviors under certain emotional states, especially negative ones, along with problematic smartphone usage. Sex differences were also observed: men displayed more sexist attitudes and violence justification, and perceived that they were more controlled by their partners. Regarding CDA behaviors, men and women showed differences in control (e.g. men considered themselves to be more controlled in terms of location and status updates), and direct aggression (e.g. men used more insults and humiliations than women). The results were discussed in terms of the importance of better understanding these risk factors to attenuate the increasing prevalence of CDA in relationships.

## Introduction

In recent years, information and communications technologies (ICTs) have served as important tools for interaction and entertainment [1–3]. This is especially the case in so-called digital generations, who socialize and interact through virtual platforms. The Internet and social networks provide tools that facilitate the creation of interpersonal relationships. These tools include making calls or video calls, sending text messages, and sharing photos, documents or location the person. Young people use many of these digital media tools for dating [4], as they facilitate communication between the members of a couple, anytime and anywhere. This is especially important in generations Y and Z (Millennials and Centennials, respectively), who consider the smartphone an indispensable device to communicate with their peers and partner. In fact, members of generation Z (born between 1995 and 2010) are referred to as ‘digital natives’, as the first generation born into an Internet-connected world [5].

However, despite the benefits of ICTs, negative effects have also been documented. In the context of relationships, smartphone usage, which allows immediate access to the Internet, and social applications and communication tools, make people more accessible and susceptible to being controlled, and more at risk of interpersonal intrusion and harassment [4,6]. In turn, this can give rise to a form of violence called partner cyber-violence or cyber-dating abuse (CDA). CDA has been defined as the ‘control, harassment, stalking, and abuse of one’s dating partner via technology and social media’ [7]. It encompasses a wide range of abusive behaviors grouped into two dimensions: direct aggression and control. Direct aggression involves behaviors aimed at harming the victim through direct attacks, such as threats, insults or spreading negative information about the partner or ex-partner, similar to identity theft. Control involves invading the privacy of a partner or surveilling their social relationships, activities, location and associates [8,9].

Research indicates that CDA is growing worldwide [6]. In particular, it is important to address this phenomenon in young adults, because the risk of intimate partner violence peaks in that age group [10], i.e., when people start to engage in more intimate and serious romantic relationships [11] and use ICTs heavily for communication [12]. Borrajo et al. [13] showed that approximately 50% of college students have been involved in CDA. In terms of severity, approximately 93% of college students perpetrated, or were victims of, minor CDA (e.g. swearing, insulting), whereas 12%-13% reported severe CDA (e.g. threats or public humiliation) [14] (for a review, see [15]). Prevalence rates of perpetration and victimization of the different types of CDA varied greatly among studies. Generally, it has been concluded that cyber-control behaviors are more frequent than direct aggression through digital tools, such as text messages, emails, mobile phone apps, messages sent through different social networks, and webcams [16]. This indicates that the less explicit nature of control behaviors means that they are more readily accepted by young people, and may be perceived as a demonstration of love or romantic jealousy [17,18].

Due to the prevalence and serious consequences of digital violence in couple relationships, obtaining more knowledge about the characteristics of victims and perpetrators, and the underlying factors, is important. Previous studies have shown a relationship between CDA perpetration and normative beliefs, violence justification [17], and sexist beliefs [19]. In addition, it has been found that being a victim of CDA is a risk factor for perpetration, and vice versa [20,21]. However, there are contradictory results about the role of other psychosocial variables including age, sex, attitudes (such as gender-based ones), and personality traits such as impulsiveness, especially in the context of problematic smartphone usage, which should be further studied to better understand CDA.

Concerning age, there is no consensus about its influence on CDA (both perpetration and victimization). While some studies found that CDA was more frequent among younger people [13], others found no such age difference [4]. This may be due to the lack of variance in age; the majority of studies included students who were only 18 years of age, with 25% being aged 19 years). Finally, another study showed opposite results, reporting that older people (> 24 years) were more likely to be victims of control [22].

The heterogeneous results on sex differences in CDA suggest that sex is an unreliable predictor of engagement in these behaviors. Regarding perpetration, some studies found that a greater proportion of women were perpetrators of control [4,13,22]. Conversely, others have shown lower levels of control and direct aggression in women [23], and higher levels of control [24] and direct aggression in men [25]. In terms of victimization, some authors concluded that women were more likely to be victims of control [4,22], while others reported a greater likelihood of men being the victim of intrusiveness, humiliation, hostility and exclusion [26,27]. Other research found that both sexes perpetrate and suffer from cyber-violence behaviors, especially control, in the context of the couple relationship [13,28]. Finally, a recent study [29] found no sex differences in the perpetration of CDA, reporting that sex was a non-significant predictor of these behaviors.

Attitudes that could lead to acceptance and justification of violence have received attention in the literature on offline dating violence [30]. According to recent data, people with attitudes that justify violence towards a partner are more likely to be perpetrators or victims of CDA in all its forms [22]. Borrajo et al. [17] found that justification of beliefs related to CDA was associated with a higher likelihood of being a direct victim of CDA, especially in women. Their study also showed that this justification was significantly linked to a higher likelihood of direct aggression in online dating relationships. Sexism has been shown to be related to aggressive behavior, and justification thereof, in offline relationships [31,32]. Individuals with sexist beliefs adopt attitudes towards others based purely on their biological sex [33]. In the context of online relationships, some research has found an effect of sexist beliefs (e.g. ‘women seek to engage men to control them) on cyberbullying behaviors against the partner [24] and CDA [34]. Specifically, Martinez-Pecino and Durán [24] found that males’ levels of hostile sexism were related to cyberbullying of their girlfriends. These authors revealed an influence of hostile sexism on cyberbullying (as a type of cyber aggression) against women in dating relationships. Rodríguez et al. [34] found that cyber aggression was positively related to sexist beliefs among boys, especially hostile ones, romantic jealousy, and other traditional forms of psychological violence. The role of sexist attitudes in being a victim of CDA has been less well-explored, although there have been some novel findings on this topic. Benevolent sexist attitudes predicted victimization involving specific forms of aggression [35]. Women who experience certain types of aggression, “under the umbrella of sexism” (p. 8), tend to minimize their situation and normalize it to some degree [35] Among other underlying cognitive mechanisms, it seems that having sexist believes could lead to minimization of the aggression exhibited (from the perpetrator perspective), as well as the aggression received (from the victim perspective) [35,36].

Regarding control behaviors, hostile sexism and other variables, such as relational offline dating violence, have been shown to predict cyber-control in boys [37]; while romantic myths and verbal-emotional offline dating violence were the main predictors of cyber-control in girls [37]. Young people, influenced by certain myths about romantic love, come to consider jealousy and control of the partner as an expression of love, which makes it difficult for them to recognize that certain behaviors are forms of violence [37–39].

Finally, one of the most important personality traits in violent behavior has been shown to be impulsivity, which is the strongest predictor of both juvenile and adult offending [40,41]. A Portuguese study carried out by Santos and Caridade [42] reported that partner self-control issues (impulsivity/aggressiveness) were among the most widely cited by adolescents as the main cause of dating violence. Impulsivity predicts online deviance, including harassing or threatening posts and illegal hacking among undergraduate students [43], and has strong, direct effects on cyberbullying among youths [44]. These authors concluded that both suffering from cyber-victimization and higher impulsivity were risk factors for becoming a cyber-aggressor. These findings suggest that direct aggression may also be the result of an impulsive reaction to a previously experienced aggressive act (reactive aggression). The widespread availability and use of smartphones can lead to impulsive decision-making. This is in line with the finding of Wilmer and Chein [45] that heavier investment of time in a mobile device is related to weaker impulse control. Furthermore, social media users have a greater tendency to prefer smaller but immediate rewards in delay-discounting tasks, which also indicates increased reward-driven impulsivity [45]. Against this background, Marcum et al. [46] found that university students with lower levels of self-control were more likely to attempt to infiltrate personal accounts and track their significant other without the knowledge of that person. Therefore, based on these findings, smartphone abuse may promote impulsive responses to, or the posting of, comments online, thereby facilitating impulsive, non-reflective communications and thus promoting CDA.

Several studies have also reported an association between impulsivity and cyber-victimization. Furthermore, impulsivity has also been related to engaging in high-risk and addictive internet behaviour. According to Álvarez-García et al. [47], impulsivity is a risk factor for being a victim of direct aggression, both directly and indirectly via its effect on high-risk internet behaviors. This result is consistent with findings indicating that low self-control is associated with increased levels of cyber victimization [44,48]. However, although the evidence about the association between impulsivity and cyber-aggression/victimization is quite clear, studies focused on this association in the dating context are scarce.

In summary, research on the role of certain factors in CDA is relatively recent and, in some cases, the data are insufficient to draw definitive conclusions. Therefore, additional research is needed to shed more light on this topic.

Because being a perpetrator or victim of cyber-aggression and cyber-control in relationships is the result of complex interactions among risk factors, the first goal of this study was to explore the diverse factors associated with CDA, including sexist attitudes and violence justification, problematic smartphone usage, and impulsiveness, as well as sex and age. No specific hypothesis was formulated given the descriptive and exploratory nature of this analysis. The second goal was to analyze differences in CDA (direct aggression and control) by sexist attitudes and violence justification, problematic smartphone usage, and impulsiveness. Related hypotheses are as follows: a) *Sexist attitudes/violence justification hypothesis*. Following previous studies, we expected that participants exhibiting sexist attitudes and violence justification would have higher CDA perpetration and victimization scores (direct aggression and control) than non-sexist participants [22]; b) *Smartphone usage hypothesis*. Based on previous findings, we expected that participants exhibiting problematic smartphone usage (e.g. dependence, significant amount of time invested, interference in daily life) would exert and receive more control and direct aggression [6,7,22]; c) *Impulsiveness hypothesis*. The most impulsive individuals were expected to display more direct aggression and control behaviors [47]. The third goal was to explore whether there were differences in the diverse range of controlling behaviors, as well as direct aggression, between women and men. According to the literature [4,22,23], there is no consensus on the utility of sex as a predictor of CDA. However, in line with previous results, we expected to observe sex differences in control behaviors and direct aggression.

## Method

### Participants

This study enrolled 697 young adults (undergraduates from various departments of the University of Jaén, Spain). The age range was 18–35 years (*M* ± *SD* = 22.08 ± 2.73 years). Participants aged between 18 and 22 years were considered as generation Z (63.8%), whereas those aged from 23 and 35 years were considered Millennials (36.2%) [49,50]. Of the participants, 548 (78.6%) were women and 149 (21.4%) were men. All participants were in, or had been in, a relationship (average duration of 31.9 months). Those not currently in a relationship were asked to think about their previous one when making their responses. The participants were recruited by emailing professors from various departments of the university. Regarding smartphone usage (number and type of apps, and time invested), most of the participants used only one instant messaging app (74.9%); use of two or three of this kind of app was less frequent (22.1% and 3%, respectively). ‘WhatsApp’ was the most commonly used application among participants. Variety was greater with respect to the social networks apps used. Most of the young adults used four (26.8%) or five (21.5%) applications, although substantial proportions also used three (17.8%) or six (15.9%). Fewer people used one or two social networks (1.9% and 5.5%, respectively), or seven (5.7%), eight (2.4%) or nine (1.7%). The most popular applications were ‘Instagram’ (92.3%), ‘YouTube’ (91.8%), ‘Spotify’ (66.6%), ‘Facebook’ (64.4%) and ‘Twitter’ (58.5%). Regarding the number of hours spent using the smartphone, during weekdays 13.1% of the participants reported using their device for an average of 1–2 hours/day, versus 37% for 2–4 hours, 27.4% for 4–6 hours, 10% for 6–8 hours, and 12.5% for more than 8 hours per day. During the weekend, the percentage of young adults who reported using their smartphone for 6–8 hours or more than 8 hours increased (14.6% and 21.1%, respectively), while the proportions of those using it less frequently decreased (10.6%, 27.8% and, 25.7% for 1–2, 2–4 and 4–6 hours, respectively).

### Measures

#### Cyber-dating abuse

*Cyber Dating Abuse Questionnaire* (CDAQ) [8]. The 40 items of this instrument measure the frequency with which a wide range of cyberbullying behaviors occurred during the last year of the couple relationship (from 1 [‘never’] to 6 [‘always: more than 20 times’]). Twenty items pertain to victimization, and twenty others to perpetration (e.g. ‘My partner or ex-partner has controlled the friendships I have on my social networks’ and ‘I have controlled the friendships of my partner or ex-partner on their social networks’). Both subscales comprise two dimensions: control (i.e., the use of electronic means to control the partner/ex-partner), and direct aggression or acting with a deliberate intention to hurt the partner/ex-partner (i.e., sending insulting and/or demeaning messages using new technologies). The total possible score ranges from 9 to 54 for control perpetration and victimization, and from 11 to 66 for direct aggression perpetration and victimization. Higher scores indicate that the individual is a bigger perpetrator or victim of control and direct aggression. Cronbach’s α is .87 for the control victimization scale, .81 for the control perpetration scale, .84 for the direct aggression victimization scale and .73 for the direct aggression perpetration scale.

#### Smartphone usage

*Dependency and Addiction to Smartphone Short-Scale* (DASS-18) [51]. This instrument consists of 18 items with 5-point Likert-type responses ranging from 1 (‘totally disagree’) to 5 (‘totally agree’). It measures the time spent using a smartphone and its apps, the anxiety felt when use is interrupted (voluntarily or involuntarily), and the extent of interference in daily life (e.g. ‘My family, partner, friends, have never complained about the time I spend looking at my smartphone’). After inverting item 7, higher average scores indicate a greater degree of dependence and addiction. The total possible score ranges from 18 to 90. Cronbach’s α is .87.

#### Impulsivity

*UPPS-P Impulsive Behavior Scale*, short Spanish version [52]. This instrument comprises 20 items that assess five impulsiveness traits (e.g. ‘I normally think carefully before doing anything’): negative urgency, lack of premeditation, lack of perseverance, sensation seeking, and positive urgency. Responses are scored on a 4-point Likert scale, ranging from 1 (‘strongly agree’) to 4 (‘strongly disagree’). The total possible score ranges from 4 to 16 for each scale. Higher scores indicate greater impulsivity. Cronbach’s α is .68 for the negative urgency scale, .78 for the lack of premeditation scale, .79 for the lack of perseverance scale, .81 for the sensation seeking scale and .61 for the positive urgency scale.

#### Sexist attitudes and violence justification

*Attitudes towards Gender and Violence Questionnaire* (AGVQ) [53]. This instrument comprises 47 statements (e.g. ‘Currently, excessive importance is being given to women victims of gender violence’) grouped into four factors: Factor 1: sexist beliefs about psychosocial differences and justification of violence as a reaction; Factor 2: beliefs about the biological utility of sexism and violence; Factor 3: conceptualization of gender violence as a private issue and inevitable problem; and Factor 4: perceptions of women’s access to economic opportunities, power and responsibility. The responses are scored on a 7-point Likert scale ranging from 1 (‘Totally disagree’) to 7 (‘Totally agree’). After reversing the scoring for the indirect items, higher scores on the first three factors indicate more sexist attitudes and greater acceptance of violence. For the fourth factor, higher scores indicate more positive perceptions. The total possible score ranges from 28 to 196 for Factor 1, 8 to 56 for Factors 2 and 3, and 3 to 21 for Factor 4. Cronbach’s α is .93 for Factor 1, .69 for Factor 2, .56 for Factor 3 and .55 for Factor 4.

#### Sociodemographic variables

Participants were also asked about their sex, age, duration of current or recent relationship, and number and type of instant messaging and social network apps used. Specifically, regarding age, we considered two groups, generation Z (18–22 years old) and Millennials (23–35 years old), in order to explore if the first generation born into an Internet-connected world differed from the second one in the variables of interest.

### Procedure

Data were collected in 2020. This was an online study; Google Forms was used to generate counterbalanced versions of the questionnaires using the D’Amato algorithm [54]. On the initial screen, participants were informed of the research goals and their rights (e.g. voluntary participation, the right to withdraw at any time and anonymity).

Regarding ethical considerations, the ‘Ethics Committee for Research with Humans of the University of Jaén’ previously approved the project research. No minors participated in the study. Informed consent was obtained at the beginning of the test; the study only proceeded if the participant checked the option ‘yes, I give my consent to participate’. The anonymity of the test and numerical coding of the responses ensured the privacy of the participants.

### Statistical analysis

Mean and standard deviation values were generated initially for the variables of interest. To achieve the first study goal, multiple correspondence analysis (MCA) was applied; this can be used to determine the underlying structure of large datasets. The MCA is a fundamental tool for analyzing relational spaces and units of analysis (categorical variables), and for illustrating and analyzing a multiplicity of relationships. In our study, there were two categorical variables (age: Z generation vs. Millennials; sex: women vs. men), and four continuous variables (total of 14 dimensions). Analyzing variables according to the levels over which the scores are distributed (low, medium, high) facilitates understanding of how they function. At the outset, MCA can be applied to quantitative and qualitative variables [55]. To transform each quantitative variable into a new categorical variable, the interquartile range was used. Thus, MCA is a simple way of graphing different types of coded variables, promoting rapid understanding and interpretation [56]. Two MCA model dimensions were used. The structure of the relationship between categories of variables was analyzed, and dimensions representing various concepts were identified. Direct aggression (as victim and perpetrator) was not included in the MCA, due to low scores and variance that prevented categorization.

To test the hypotheses associated with the second goal of this study, Student’s t-tests were performed. Perpetration and victimization of control and direct aggression were considered as dependent variables. The variables were dichotomized into low and high levels for high discriminability; their associations with the main outcome variable were then assessed. Specifically, the following variables were analyzed: high dependence and addiction to the smartphone (DASS-18), negative urgency, positive urgency (UPPS-P), sexist beliefs about psychosocial differences and justification of violence, beliefs about the biological utility of sexism and violence, and conceptualization of gender violence as a private issue and inevitable problem (AGVQ). Finally, to explore the third goal of this study, i.e., the effect of sex on the likelihood of being the perpetrator or victim of control and direct aggression was explored in depth through an item analysis. In both analyses, a critical α level of 0.05 was used for all comparisons. Additionally, Holm–Bonferroni sequential correction was used to decrease type-1 error in the case of multiple comparisons. Holm’s adjusted alpha level was calculated via an Excel calculation form by Gaetano [57].

## Results

### Descriptive analysis

The mean and standard deviation data are shown in Table 1. Focusing on the main outcome variable (CDA), it can be seen that average scores for direct aggression in the context of the couple relationship were especially low, from both the victim and perpetrator perspective (score range: 11–66).

**Table 1:** Descriptive statistics for the dimensions of each variable of interest.

Variable	Dimensions (subscales)	*M*	*SD*
*Smartphone usage*	Problematic smartphone usage	2.56	0.74
*Impulsivity*	Negative urgency	9.61	3.12
	Positive urgency	9.35	2.45
	Sensation seeking	9.60	3.09
	Lack of premeditation	9.36	1.94
	Lack of perseverance	7.69	3.17
*Gender and violence attitudes*	Sexist beliefs and violence justification	35.87	13.72
	Beliefs about the utility of sexism and violence	19.19	8.01
	Conceptualization of gender violence as inevitable and private	14.79	5.58
	Perception of women’s access to power and responsibility	15.38	4.25
*Cyber-dating abuse*	Victim of control	15.76	8.84
	Victim of direct aggression	12.25	3.56
	Perpetrator of control	15.28	8.14
	Perpetrator of direct aggression	11.77	2.23

### Multiple correspondence analysis

**Fig 1:**
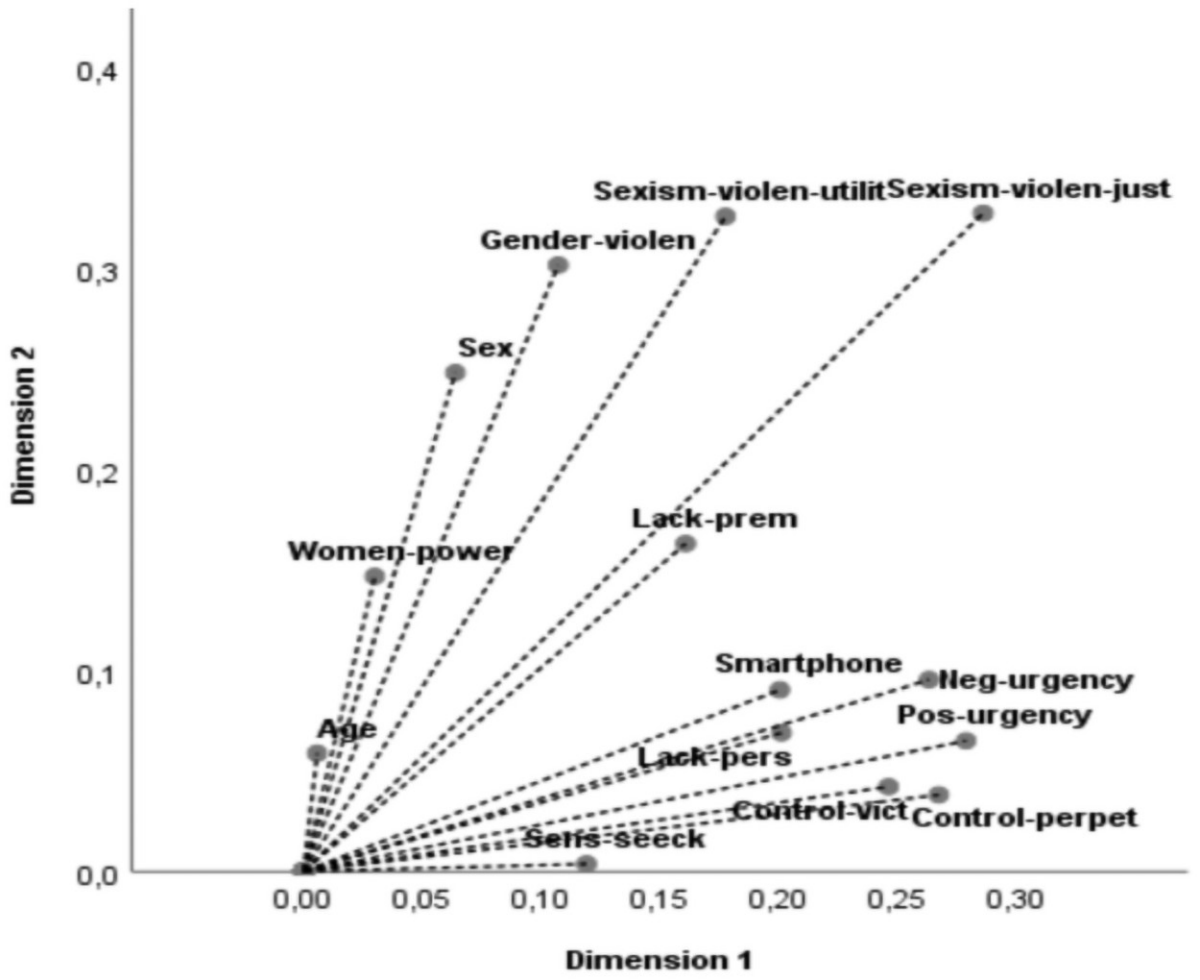
Discrimination indices of the variables for D1 and D2. *Variables and abbreviations: (a) Problematic smartphone usage = smartphone; (b) Sexist beliefs and violence justification = sexism-violen-just; (c) beliefs about the utility of sexism and violence = sexism-violen-utilit; (d) Conceptualization of gender violence as inevitable and private = gender-violen; (e) Perception of women’s access to power and responsibility = women-power; (f) Negative urgency = neg-urgency; (g) Positive urgency = pos-urgency; (h) Sensation seeking = sen-seeck; (i) Lack of premeditation = lack-prem; (j) Lack of perseverance = lack-pers; (k) Victim of control = control-vict; (l) Perpetrator of control = control-perpet.

The MCA model, which captured the distribution and categories of the variables, showed satisfactory reliability for both dimensions (D1: α = .63, D2: α = .53). These reliability values are in the optimal range for this type of analysis [58]. The distribution of the points in the factorial space of the categories indicated acceptable inertia (D1 inertia = .172, D2 inertia = .141). Controlling behaviors, from both the victim and perpetrator perspectives, had a higher saturation index for D1. Problematic smartphone usage, and factors associated with impulsivity, especially urgency to act under negative and positive emotions, were also discriminated more in D1. D2 included participants’ sex and the following attitudinal variables: sexist beliefs and justification of violence, beliefs about the utility of sexism and violence, and conceptualization of gender violence as inevitable and private. Meanwhile, age, lack of premeditation, sensation seeking, and perception of women’s access to power and responsibility showed low levels of saturation (Table 2, Fig 1).

**Table 2:** Ability of the variables to discriminate between the two dimensions.

		Dimension		Mean
		1	2	
*Smartphone usage*	Problematic smartphone usage	**.201**	.090	.145
*Gender and violence attitudes*	Sexist beliefs and violence justification	.286	**.327**	.307
	Beliefs about the utility of sexism and violence	.178	**.326**	.252
	Conceptualization of gender violence as inevitable and private	.107	**.302**	.204
	Perception of women’s access to power and responsibility	.030	**.147**	.088
*Impulsivity*	Negative urgency	**.263**	.095	.179
	Positive urgency	**.279**	.065	.172
	Sensation seeking	**.119**	.004	.061
	Lack of premeditation	**.161**	**.163**	.162
	Lack of perseverance	**.201**	.069	.135
*Cyber-dating abuse*	Victim of control	**.246**	.042	.144
	Perpetrator of control	**.267**	.038	.153
*Sex*		.064	**.248**	.156
*Age*		.006	.059	.032
	Total active	2.408	1.976	2.192
	% of variance	17.200	14.112	15.656

The multifactorial analysis organized the categories into quadrants in the factorial plane. Closer proximity on an axis indicates a stronger association among variables, while those located further away from each other have weaker associations.

**Fig 2:**
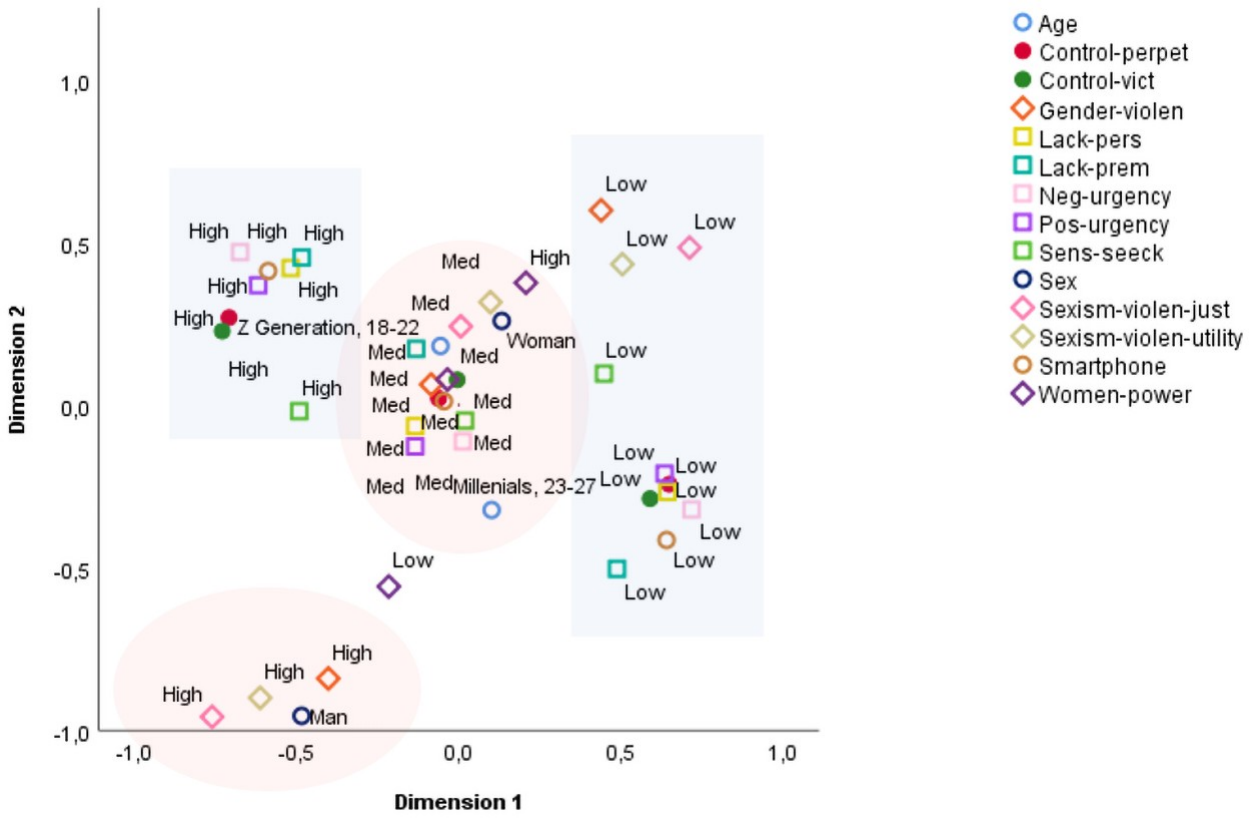
Diagram showing the distribution of the variables in a two-dimensional map.

Higher scores for perpetration of control behaviors were associated with a high level of victimization, that is, participants tended to exercise as much control over their partners as was exercised over themselves. High scores for the impulsivity traits, especially negative and positive urgency, were located close together on the plane (with high discriminatory power). The highest scores for problematic smartphone usage were in the same plane. On the contrary, low perpetration and victimization, low impulsivity (mainly reflected in control under negative emotions), and low problematic smartphone usage were grouped. Although further apart in vector space, the lowest levels of sexist attitudes and violence justification (except the perception of women’s access to power and responsibility) were in the same explanatory dimension as cyber-control behaviors (Fig 2).

The two categories of sex (women and men) were on opposite planes in vector space, as were the categories of the other variables adjacent to women and men. Regarding the attitudinal variables included in the model (sexist attitudes and violence justification), in men there was an association with strong sexist beliefs and violence justification, beliefs about the utility of sexism and violence, and conceptualization of gender violence as a private and inevitable problem. The medium levels of the variables were located near to the central part of the axes and formed a compact set of associations, i.e., the four factors of the attitudinal variables (sexist beliefs and violence justification), dependence and addiction to the smartphone, perpetration and victimization of control, and the four factors of impulsivity. The ‘woman’ category is enclosed within this set of medium-level variables, located near to the center of the two-dimensional space.

With the lowest discriminatory power, the two age cohorts in this study were allocated to the central part of the vector space; thus, age was not a particularly relevant discriminatory variable associated with CDA. Nevertheless, the MCA allowed us to explore the role of age. The oldest participants (Millennials) were located between the low problematic smartphone usage, low and medium impulsivity traits, and low and medium victimization and perpetration of control. Meanwhile, being younger (Z generation) was associated with a medium level of control perpetration and victimization, as well as medium levels of the attitudinal variables and impulsiveness.

### Comparison of mean values of the dependent variables: CDAQ

The t-tests revealed greater perpetration and victimization of control and direct aggression in those with high levels of problematic smartphone use, and sexist beliefs about psychosocial differences and justification of violence. Participants high in negative urgency were more controlling of their partner, and were also subject to more control and direct attacks than those who scored low on this variable. Regarding positive urgency, the results showed that the highest levels were associated with more control behaviors and direct aggression, as well as a perception of being the victim of direct aggression (Table 3).

**Table 3:** Descriptive and t-test results for cyber-dating abuse (control and direct aggression) from the victim and perpetrator perspectives.

		Control	Direct aggression	Control	Direct aggression
		Victim’s perspective		Perpetrator’s perspective	
Smartphone usage	*t* (gl); *p*	-5.37 (320.3); < .05*	-2.61 (347); < .05*	-5.73 (324.9); < .05*	-1.97 (359.2); = .05*
	*M*_low_ (*SD*)	13.82 (7.63)	11.97 (3.80)	13.57 (7.16)	11.64 (2.18)
	*M*_high_ (*SD*)	19.07 (10.72)	13.11 (4.56)	18.73 (9.81)	12.11 (2.36)
Negative urgency	*t* (gl); *p*	-2.30 (373.5); < .05*	-2.53 (361.9); < .05*	-2.01 (377.0); = .05*	-1.65 (376.9); >.05
	*M*_low_ (*SD*)	14.96 (8.29)	11.87 (2.93)	14.65 (8.02)	11.70 (2.21)
	*M*_high_ (*SD*)	17.13 (10.01)	12.77 (3.95)	16.38 (8.71)	12.09 (2.39)
Positive urgency	*t* (gl); *p*	-1.69 (440.5); >.05	-1.93 (405.1); = .05*	-2.64 (414.8); < .05*	-2.16 (400); < .05*
	*M*_low_ (*SD*)	15.10 (8.11)	11.85 (2.84)	14.38 (7.13)	11.58 (1.88)
	*M*_high_ (*SD*)	16.46 (9.36)	12.45 (3.78)	16.37 (9.12)	12.02 (2.55)
Sexist beliefs and violence justification	*t* (gl); *p*	-3.36 (334); < .05*	-2.85 (292.3); < .05*	-3.40 (366); < .05*	-3.39 (268.2); < .05*
	*M*_low_ (*SD*)	14.40 (7.69)	11.73 (2.60)	13.74 (7.23)	11.39 (1.52)
	*M*_high_ (*SD*)	17.54 (10.00)	12.78 (4.24)	16.47 (8.23)	12.21 (2.86)
Conceptualization of gender violence as inevitable and private	*t* (gl); *p*	-1.25 (378); >.05	-1.17 (378); >.05	-0.61 (378); >.05	-0.54 (378); >.05
	*M*_low_ (*SD*)	15.41 (8.54)	12.11 (3.62)	15.01 (8.65)	11.81 (2.18)
	*M*_high_ (*SD*)	16.52 (8.78)	12.59 (4.29)	15.51 (7.33)	11.93 (2.37)
Beliefs about the utility of sexism and violence	*t* (gl); *p*	1.73 (404); >.05	1.26 (393.6); >.05	0.75 (404); >.05	-0.90 (404); >.05
	*M*_low_ (*SD*)	16.05 (9.26)	12.47 (4.27)	15.41 (8.46)	11.71 (2.06)
	*M*_high_ (*SD*)	14.55 (7.93)	12.02 (2.93)	14.80 (7.79)	11.92 (2.60)

The differences in problematic smartphone use levels on perpetration and victimization of control, as well as differences in sexist beliefs about psychosocial differences and justification of violence levels on perpetration and victimization of control and perpetration of direct aggression survived the Holm-Bonferroni correction (*p* < .0025).

### Sex-related differences in CDAQ: An item analysis

The t-test showed that men scored higher on cyber-control victimization compared with women, *t*(695) = 2.28, *p* < .05, *M*_men_ = 17.21, *SD*_men_ = 8.95, *M*_women_ = 15.36, *SD*_women_ = 8.78. There were no sex differences in the other CDA subdimensions.

In-depth study of the sex differences showed that, regarding direct aggression, women and men showed differences with respect to acting with the deliberate intention to hurt the partner through smartphones and social networks. Women are more likely to be perpetrators of threats to spread secrets or embarrassing information using new technologies, whereas men are more likely to act against their partner in terms of the posting of music, poems, phrases, etc., on a social networking site with the intent to insult or humiliate. No sex differences were found in the context of victimization. Regarding control, men were more likely to perceive their partners to be monitoring their friends on social networks, where they are and with whom, and their status updates on social networks. Men also perceived that they were threatened to answer calls or messages immediately more often than women. No sex differences were found in the context of perpetration (Tables 4 and 5). Notably, only sex differences in threatening to answer calls or messages immediately using new technologies survived the Holm-Bonferroni correction for multiple testing (*p* < .028).

**Table 4:** Sex-related differences for each item of the direct aggression subscale, for both victimization and perpetration.

			Victimization					Perpetration				
Item	Description item	Sex	*M*	*SD*	*t*	*gl*	*p*	*M*	*SD*	*t*	*gl*	*p*
2	Threats through new technologies to physically harm	Men	1.07	0.43	0.57	695.00	.57	1.04	0.20	1.71	187.14	.09
		Women	1.05	0.35				1.01	0.13			
3	Creating a fake profile on a social network to cause problems	Men	1.05	0.45	0.97	695.00	.34	1.05	0.36	0.77	695.00	.44
		Women	1.03	0.27				1.03	0.25			
4	Writing a comment on the wall of a social network to insult or humiliate	Men	1.10	0.53	1.04	179.19	.30	1.11	0.59	1.03	177.40	.30
		Women	1.05	0.32				1.05	0.35			
6	Spreading secrets and/or compromised information using new technologies	Men	1.21	0.69	1.42	208.87	.16	1.09	0.48	0.86	194.21	.39
		Women	1.12	0.58				1.05	0.35			
8	Threatening to spread secrets or embarrassing information using new technologies	Men	1.08	0.49	-0.35	695.00	.73	1.01	0.08	-2.13	685.77	.03*
		Women	1.10	0.51				1.03	0.26			
9	Using new technologies to pretend to be me/my (ex) partner and create problems	Men	1.02	0.18	-0.56	695.00	.58	1.03	0.26	0.91	156.83	.36
		Women	1.03	0.30				1.01	0.09			
10	Sending insulting and/or demeaning messages using new technologies	Men	1.37	0.96	0.43	695.00	.67	1.16	0.49	-1.59	394.24	.11
		Women	1.33	0.98				1.25	0.82			
12	Sending and/or uploading photos, images and/or videos with intimate or sexual content without permission	Men	1.01	0.16	-0.56	695.00	.58	1.00	0.00	-0.74	695.00	.46
		Women	1.03	0.29				1.00	0.06			
15	Pretending to be another person using new technologies to test a partner	Men	1.16	0.66	1.49	179.90	.14	1.10	0.48	0.64	695.00	.52
		Women	1.08	0.41				1.08	0.38			
16	Posting music, poems, phrases, etc. on a social networking site with the intent to insult or humiliate	Men	1.34	0.95	1.23	210.49	.22	1.36	0.93	2.39	188.14	.02*
		Women	1.24	0.81				1.16	0.64			
18	Spreading rumors, gossip and/or jokes through new technologies with the intention of ridiculing	Men	1.11	0.59	-0.22	695.00	.83	1.05	0.27	0.11	695.00	.91
		Women	1.13	0.59				1.04	0.31			

**Table 5:** Sex-related differences for each item of the cyber-control subscale, for both victimization and perpetration.

			Victimization					Perpetration				
Item	Description item	Sex	*M*	*SD*	*t*	*gl*	*p*	*M*	*SD*	*t*	*gl*	*p*
1	Controlling status updates on social networks	Men	2.30	1.60	2.01	221.61	.05*	1.99	1.30	-0.35	261.19	.73
		Women	2.00	1.48				2.04	1.48			
5	Using passwords (phone, social networking, email) to browse messages and/or contacts without permission	Men	1.32	0.89	-0.52	695.00	.61	1.31	0.83	-1.06	260.18	.29
		Women	1.36	0.93				1.39	0.94			
7	Checking the last connection in mobile applications	Men	2.75	1.65	1.70	695.00	.09	2.61	1.53	1.12	695.00	.26
		Women	2.50	1.62				2.45	1.59			
11	Checking social networks, WhatsApp or email without permission	Men	1.55	1.08	0.85	695.00	.39	1.36	0.85	-1.70	265.80	.09
		Women	1.47	1.02				1.49	0.99			
13	Using new technologies to control where you are/I am and with whom	Men	1.97	1.48	2.15	216.38	.03*	1.85	1.35	0.45	695.00	.65
		Women	1.68	1.33				1.79	1.37			
14	Threatening to answer calls or messages immediately using new technologies	Men	2.15	1.52	2.99	216.23	.00*	1.60	1.21	0.13	695.00	.90
		Women	1.74	1.35				1.59	1.22			
17	Checking a partner’s mobile phone without permission	Men	1.63	1.16	1.05	216.54	.29	1.44	0.93	-1.25	258.98	.21
		Women	1.52	1.04				1.55	1.04			
19	Excessive calls to control where you are/I am and with whom	Men	1.49	1.11	1.53	219.07	.13	1.29	0.84	0.14	695.00	.89
		Women	1.34	1.01				1.28	0.91			
20	Controlling friends on social networks	Men	2.06	1.44	2.31	230.07	.02*	1.79	1.31	0.48	695.00	.63
		Women	1.34	1.01				1.28	0.91			

## Discussion

In the present study, we first aimed to determine the associations of variables related to attitudes (sexist attitudes, violence justification), personality traits (impulsiveness) and problematic smartphone use (dependency and addiction) with control and direct aggression behaviors towards the partner. The associations of age and sex with CDAQ scores were also analyzed. Second, differences in cyber-dating abuse according to the variables most strongly associated (in the MCA) with this type of behavior were analyzed. Finally, sex differences were explored, i.e., the behaviors specifically displayed by men and women were identified.

The descriptive analysis performed previously showed that the prevalence of cyber-control behavior was higher than that of cyber-aggression. This finding is in line with other research on this topic [8,22], and is supported by the justification and normalization of control behaviors usually seen in young people. A study by de Miguel [59] found that one in three young people consider control of the partner to be an inevitable behavior, and that controlling behavior proves one’s love and trust of the partner.

The MCA allowed us to determine the multiple associations among several variables, and the extent to which they were clustered together on the axial map (first study goal). The distributions of the variables on the plot indicated that the highest levels of victimization and perpetration of control were closely associated, suggesting that being the victim of and performing these types of behaviors co-occur within the same couple. Víllora et al. [20,21] previously found that being a victim of CDA was a risk factor for perpetration, and vice versa. The map of the variables described above could explain this result: controlling behaviors were linked with impulsivity traits, particularly the tendency to lose control in the context of both negative and positive emotions. In addition, problematic smartphone usage was linked to control of one’s partner. In the opposite plane, the lowest levels of victimization and perpetration of control (which also appeared together) were closely associated with the capacity to manage behaviors under the influence of negative and positive emotional states. Regarding age, although the results revealed that age is not particularly relevant to control behaviors, there was a tendency toward some differences between Z generation and Millennials. While the youngest participants showed medium or high levels of control, the oldest tended to show medium or low levels of controlling behaviors. The same pattern was seen for smartphone usage and impulsivity traits. The results obtained by previous studies are heterogeneous, which makes drawing definitive conclusions difficult. Some authors found no differences in CDA among university students according to age [4,60,61]. However, other studies showed that the prevalence of CDA increases as age decreases [17], although in one report a higher likelihood of being the victim of control was seen with increasing age [6,22], and in another the prevalence of CDA was lower in older couples [62]. Most of the research reviewed focused on a specific age range, and was conducted from a developmental perspective, thereby mainly including adolescents or young people, or both. Considering generational cohorts from a “technological” perspective, due to the extensive influence of technologies on communication and interactions, is another important consideration. Interpreting online behaviours and, specifically, CDA in this context could be useful [63]. In this regard, our results pertaining to age are valuable given the scarcity of data on the association of age with CDA.

The position of the sex variable on the map indicated that women were most likely to show medium levels of control, both as recipients and actors. However, female participants scored lower for sexist attitudes and violence justification than men. Overall, the inability to control behavior under the influence of both positive (e.g. not thinking about the consequences of one’s actions when feeling happy or encouraged) and negative emotional states (e.g. acting without thinking when feeling irritated), along with problematic smartphone usage (excessive time invested, interference with daily life and anxiety when the device cannot be used), were important risk factors. Meanwhile, being able to control behavior in the context of certain emotional states and healthy smartphone usage were protective factors. As concluded by some other authors, the inability to regulate emotions and delay responses under their influence can lead to behaviors that offend others [40,41], even including one’s own partner [42]. Impulsivity could also explain, in part, why being a victim and being a perpetrator appear together. As Vazsonyi et al. [44] suggested, cyber-aggression could be the result of an impulsive reaction to a previously experienced aggressive act.

The findings on differences in CDA according to individual characteristics supported and extended the above-mentioned results (second study goal). Participants with a greater tendency to lose control under positive and negative emotional states scored higher for perpetration of control and direct aggression. These results add to the scarce data on the importance of impulsivity to CDA [47]. Impulsive individuals would likely have problems in managing their impulse to control their partner, due to difficulty in thinking about the consequences before acting when they feel anxious, angry, or even excited. Furthermore, cyberspace may be an environment that encourages people to act rashly, without thinking. Finally, as previous literature has shown, there is a link between impulsivity and problematic smartphone usage [64,65]. In summary, a person who shows intense smartphone use (i.e., to communicate with their partner) experiences craving when its use is not possible, which is associated with difficulties in managing his or her behavior under positive or negative emotions; this could lead to controlling, or even offensive, behavior toward the partner through the smartphone.

Our results also showed that individuals with greater dependence on smartphones scored higher for victimization and perpetration of control and direct aggression, which confirms that the smartphone can be used as a tool to control, and even offend, the partner. These findings agree with those of Víllora et al. [22], who found that mobile phone misuse was associated with higher levels of perpetration and victimization of control and direct aggression. Other studies also concluded that greater use of technological devices [8] and problematic Internet use [66] are associated with greater levels of CDA.

Sexist attitudes and violence justification showed a moderate association with CDA in this study; however, comparison of the mean values of participants with the lowest and highest levels of this attitudinal variable revealed significant differences. Participants who had stronger sexist beliefs (e.g. men are superior at performing the tasks that they have traditionally been associated with; women should continue to perform tasks that have been traditionally considered as feminine) and violence justification (e.g. the victim is responsible) presented along with higher levels of victimization and perpetration of both control and direct aggression. In addition, those who considered sexism impossible or difficult to overcome due to human nature and biological gender differences felt more controlled by their partner. Regarding smartphone use, as these devices provide access to the Internet, social networks and communication apps anywhere and at any time, they have become a means for the expression of sexist attitudes. Moreover, the features of ICTs and their potential for enhancing socializing could make smartphones in particular a vehicle for expressing sexism [67], such as hostile sexism [68]. Therefore, overuse of this device could lead to increases in sexist attitudes since [69], as we said above, it seems to facilitate the subtle expression of certain controlling behaviors.

In line with the above-mentioned results, as men scored higher for sexist attitudes and violence justification, one would expect them to perpetrate more CDA [19,24,69]. However, in terms of cyber-control victimization, male participants perceived themselves as being more controlled by their partners. The level of sexism, along with other factors not evaluated in this study, likely played a role in this finding. While male participants showed sexist attitudes, they could rationalize their own control behaviors, and were also more aware of being the recipients thereof [70]. These results may indicate a difference in the perception and interpretation of CDA behaviors between women and men. In the literature, there is no consensus regarding sex differences in the prevalence of CDA. In accordance with our results, Bennett et al. [26] also found that men more frequently report being the victim of online intrusion from their partners than women, as well as hostility, humiliation and exclusion, which could all be classified as forms of direct aggression. In the same vein, Kellerman et al. [27] found that men reported higher levels of online victimization, although they did not specify the category of CDA. Other studies found no sex differences in control or direct aggression [25], although in one study women were more likely to be the victims of control [4]. The key to the heterogeneity in these results may lie, as we pointed out, in the mediating role of sexism and violence justification.

To further explore differences not only in the quantity, but also the form, of CDA between the sexes, a CDAQ item analysis was performed (third study goal). Regarding specific behaviors related to direct aggression, women were more likely to threaten to spread secrets or embarrassing information through ICTs, whereas men were more likely to post music or phrases on social networks aimed at insulting or humiliating their partner. Regarding control behaviors, men felt more controlled than women. This control is perceived by men in the context of social networks, i.e., in terms of their friends and status updates. Men also reported more pressure to answer calls or messages immediately. As illustrated by the above, men and women differ specifically in terms of the degree to which they feel controlled by their partners, with men showing higher scores. Previous studies have also analyzed possible sex differences in the various behaviors captured by the questionnaires used in this study, such as that of Piquer et al. [28]; however, that study only reported frequency data; statistical analysis was not carried out to compare the sexes.

The present study had some limitations that necessitate caution when interpreting the results. First, some results did not survive to the Holm-Bonferroni correction. This method deals with familywise error when conducting multiple hypothesis tests. However, it substantially reduces statistical power and thus the probability of detecting significant effects, thereby increasing Type II errors [71]. Second, studies on psychological factors that use self-report measures may be affected by social desirability bias. This is particularly relevant for variables that capture morally reprehensible behaviors or attitudes, such as CDA. It is assumed that the measures used in the study were developed under the assumption that some respondents may consciously or unconsciously under-report undesirable behaviors. To minimize social desirability bias, psychometric analysis of the self-report measures was performed, as well as both validation and reliability tests. To deal with social desirability, in future research both CDA and reaction time-based measures, such as the implicit association test (IAT) [72,73] and priming tasks [74], could be used together to direct and indirectly assess the construct. Third, the homogeneity of the sample (all university students), analysis of a single culture, and greater number of women may reduce the validity and generalizability of the results. Fourth, this study focused on current and past relationships, so possible memory biases in the second condition were not controlled for and may have affected the participants’ responses. Finally, we cannot rule out random responding because questions designed to assess attention were not included. Thus, in future research it would be also interesting to explore this topic in non-university couples, as well as to include more men in the sample. In addition, only some risk factors were analyzed in this study. Exploring the role of other variables in CDA could lead to more accurate characterization of perpetrators and victims of control and direct aggression. In the same vein, it is also important to uncover the psychological motivations for perpetrating CDA, for earlier detection of CDA and more effective educational programs to prevent this increasingly prevalent phenomenon.

Despite its limitations, the present study provides novel and specific information about the variables associated with CDA. First, it was found that smartphones are often used to control the partner, although the incidence of cyber-aggression was overall very low. Second, the results allowed us to create a “map” of risk factors. Specifically, control behavior perpetrators are likely to show a greater degree of problematic smartphone use, a tendency to lose control in the context of positive and negative emotions, sexist beliefs about psychosocial differences, and violence justification. The same factors were also associated with being a victim of CDA. Perpetration of direct aggression was also associated with impulsivity (negative and positive urgency) and the abovementioned sexist beliefs. Therefore, to prevent and minimize these types of cyber behaviors in the relationships, identifying the sexist beliefs and romantic myths that contribute to the perception of some control behaviors being normative in the context of a romantic relationship seems important [36]. Moreover, as the smartphone can be used for control and surveillance [3,75,76], and because problematic use thereof has been shown to be linked to CDA, educational programs promoting healthy smartphone use are needed. Some aspects of impulsivity were linked with a propensity to control the partner in this study. Therefore, skills promoting the control of behavior in the context of certain emotional states, especially negative ones, may be crucial to mitigate negative effects.
